# Time to symptom onset and manual reduction outcomes as predictors of bowel viability in incarcerated obturator hernias

**DOI:** 10.1038/s41598-024-65375-9

**Published:** 2024-06-21

**Authors:** Yusuke Gokon, Yusuke Ohki, Takahiro Ogino, Keiichiro Hatoyama, Kenji Shimizu, Kazunori Katsura, Toshiaki Kashiwadate, Takayuki Abe, Koichiro Sato

**Affiliations:** Department of Surgery, Iwate Prefectural Iwai Hospital, 17 Odaira, Kozenji, Ichinoseki, 029-0131 Japan

**Keywords:** Obturator hernia, Manual reduction, Bowel viability, Incarceration duration, Surgery, Predictors, Gastrointestinal diseases, Digestive signs and symptoms, Risk factors

## Abstract

The current study aimed to identify the indications for manual reduction in incarcerated obturator hernias (OH). Further, whether time to symptom onset and manual reduction outcomes can be predictors of bowel viability and the need for bowel resection in incarcerated OH were examined. This retrospective study included 26 patients with incarcerated OH who underwent surgery. All patients underwent manual reduction, and computed tomography scan after manual reduction confirmed hernia release. Multivariate analyses were performed to determine the predictors of bowel resection. The bowel resection group had a significantly longer average time to symptom onset than the nonbowel resection group (88 vs 36 h). Further, the bowel resection group was more likely to have failed manual reduction than the nonbowel resection group. A time to symptom onset of ≥ 72 h and failed manual reduction were significant predictors of bowel viability. Age, sex, hernia localization, American Society of Anesthesiologists physical status score, and laboratory findings did not differ significantly between the bowel resection and nonbowel resection groups. Time to symptom onset and manual reduction outcomes are significant predictors of bowel viability in incarcerated OH. Patients with a time to symptom onset of ≥ 72 h and failed manual reduction require surgical evaluation due to a high risk of bowel nonviability. Therefore, a cautious approach is required in the management of OH, and further research on optimized treatment protocols should be conducted.

## Introduction

Obturator hernias (OHs) are relatively rare, accounting for 0.07%–1% of all hernia cases^1^. Emergent surgery is a common approach for incarcerated OH. Recently, some reports have evaluated the use of elective surgery after noninvasive manual reduction^2–5^. Manual reduction is the initial approach for incarcerated OH, followed by elective surgery, at our institution. Previous research has reported the usefulness of manual reduction for incarcerated OH^6^. However, a consensus regarding the indications for manual reduction in incarcerated OH has not been established^5^. In inguinal and femoral hernia guidelines, a time to symptom onset of ≥ 24 h is considered a useful predictor of nonviable bowel strangulation^7^. Previous reports have revealed that the criteria for incarcerated OH are less likely to be severe than those for incarcerated inguinal hernias because OH commonly involves a part of the small bowel circumference only, which is referred to as Richter-type hernia. However, a specific time to symptom onset was not identified as only a small number of cases were evaluated^5,8^. The current study aimed to validate the indications for manual reduction in incarcerated OH.

## Methods

This study analyzed 26 patients with incarcerated OH who underwent surgery from 2017 to 2023 at Iwate Prefectural Iwai Hospital. All patients were diagnosed with incarcerated OH on computed tomography (CT) scans. Manual reduction was the initial approach for incarcerated OH, followed by elective surgery after approximately 1 month^6^. If the hernia was irreducible, emergent surgery was performed. The clinical characteristics and operative outcomes between patients with or without bowel resection were retrospectively compared. Postoperative complications were defined as adverse events occurring within 30 days of surgery or during hospitalization. The severity of complications was assessed using the Clavien–Dindo classification (grades I–V)^9^.

The study protocol was approved by the institutional ethics committee or review board (accession no. Iwai R4–1078). Informed consent was obtained from all participants.

### Manual reduction maneuver

All patients underwent ultrasound-guided manual reduction of OH^6^. The patients were placed in the supine position with the leg in abduction, flexion, and external rotation. The surgeon visually recognized the incarcerated OH at the anterior side of the adductor longus muscle on ultrasonography. Subsequently, manual reduction was performed by compressing the posterior side of the adductor longus muscle and the lateral side of the labium majus manually until the hypoechoic mass disappeared. If the incarcerated hernia was not released promptly, the surgeon’s assistant bent and stretched the patient’s leg repeatedly^3,4,10^. Thereafter, the patient underwent CT scan to confirm the release of the hernia and the absence of perforation.

### Surgical procedure

If manual reduction was successful, the suprapubic midline extraperitoneal approach was used in elective surgery^11^. A 4–6 cm suprapubic vertical incision was made, and the space of Retzius was dissected. Then, the obturator hernia was inverted under direct view. Conversely, a lower midline laparotomy was utilized in emergent surgery.

### Statistical analysis

All statistical analyses were performed using JMP Pro version 15 with the Student’s *t*-test and the Fisher’s exact test. A P value of < 0.05 was considered statistically significant.

### Ethical approval

All procedures performed in this study involving human participants were in accordance with the ethical standards of our institutional research committee and with the 1964 Helsinki declaration and its later amendments or comparable ethical standards.

### Informed consent

Informed consent was obtained from all study participants.

## Results

**Figure 1:**
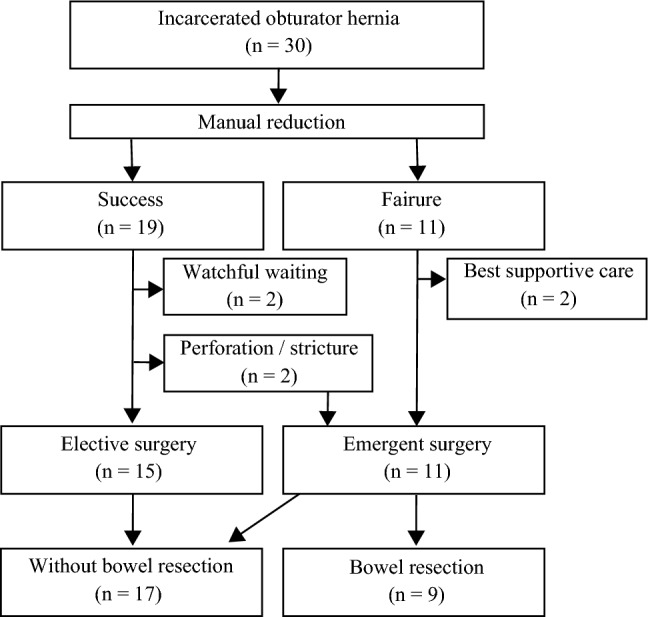
Flowchart of patients. In total, 30 patients underwent manual reduction. The procedure was successful in 19 patients, of whom two opted for watchful waiting. Of 19 patients, 17 underwent elective surgery. However, two patients underwent emergent surgery in the waiting period because of late-onset constriction and small bowel perforation. In total, 11 patients had failed manual reduction, and nine patients underwent emergent surgery. Two patients received the best supportive care. Eventually, 17 patients did not undergo bowel resection, and nine patients underwent the procedure.

**Figure 2:**
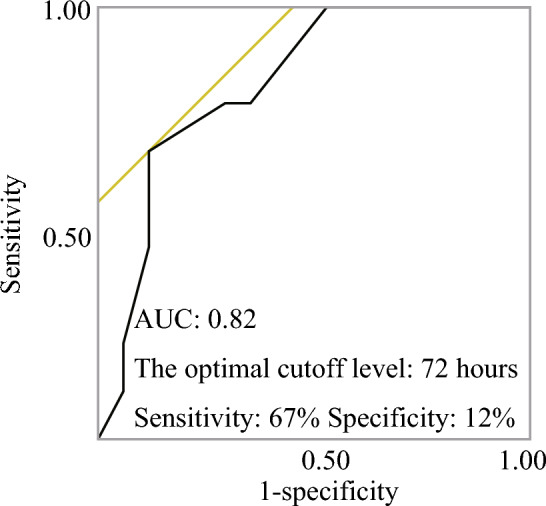
The receiver operating characteristic curves for predicting poor bowel viability. The optimal cutoff levels for a time to symptom onset of 72 h, with a sensitivity of 67%, specificity of 12%, and area under the curve (AUC) of 0.82.

Figure 1 shows the patient flowchart. In total, 30 patients underwent manual reduction. In 19 cases, manual reduction was successfully performed. Further, two patients wanted vigilant observation owing to chronic pulmonary insufficiency. Of 19 patients, 17 underwent elective surgery. However, during the interim, two patients required emergent surgical intervention due to the development of late-onset constriction and small bowel perforation. In total, 11 patients had an unsuccessful manual reduction. Among them, nine underwent urgent surgical procedures. Two patients with deteriorating health conditions received palliative care alone, and they died the following day. Eventually, 17 patients did not undergo bowel resection, and nine patients required the procedure. The patients who underwent surgery were divided into the bowel resection and nonbowel resection groups (Table 1). The average time to symptom onset of the bowel resection group was significantly longer than that of the nonbowel resection group (88 vs 36 h, P = 0.03). The bowel resection group was more likely to experience failed manual reduction than the nonbowel resection group (P = 0.001). No significant differences were observed in terms of age, sex, hernia localization, American Society of Anesthesiologists physical status (ASA-PS) score, laboratory findings, and postoperative complications between the bowel resection and nonbowel resection groups. Two patients in the nonbowel resection developed postoperative complications, particularly ileus and tachycardia. The perioperative mortality rate of the bowel resection and nonbowel resection groups was 0%. The receiver operating characteristic curves for predicting poor bowel viability and the optimal cutoff levels for time from onset were 72 h, for which the sensitivity was 67%, specificity was 12%, and area under the curve (AUC) was 0.82 (Fig. 2). Table 2 shows the characteristics of patients with bowel resection and those without compared via univariate and multivariate analyses. A time to symptom onset of ≥ 72 h and failed manual reduction were significant factors in the multivariate model (P = 0.03, 0.005, respectively).

**Table 1:** Characteristics of patients with and without bowel resection.

Clinicopathological features	Patients who did not undergo bowel resection n = 17 (%)		Patients who underwent bowel resection n = 9 (%)		P value
Age (years)†	84.6	(6.9)	88.0	(6.4)	0.23
Sex					
Male	0	(0)	1	(11)	0.34
Female	17	(100)	8	(89)	
Hernia localization					
Right	10	(59)	4	(44)	0.66
Left	7	(41)	5	(56)	
Manual reduction					
Failure	2	(12)	7	(78)	0.001*
Success	15	(88)	2	(22)	
Time to symptom onset (hours)†	36.1	(52.4)	88.0	(61.2)	0.03*
ASA-PS score					
2	5	(29)	3	(33)	1.00
3	12	(71)	6	(67)	
Laboratory findings					
White blood cell count†	10,557	(4677)	10,270	(3500)	0.87
C-reactive protein level (mg/dL)†	1.2	(3.0)	3.5	(5.1)	0.17
Lactate level (mmol/L)†	1.2	(0.51)	1.1	(0.27)	0.77
Postoperative complications					
With	15	(88)	9	(78)	0.52
Without	2	(12)	0	(22)	

*Statistical significance. The Student’s *t*-test was used to assess age, time to symptom onset, and laboratory findings. The Fisher’s exact test was utilized to evaluate other parameters. Values in parentheses are percentages unless indicated otherwise. † values are mean (SD).

**Table 2:** Characteristics of patients with bowel resection and those without compared via univariate and multivariate analyses.

Covariates	Univariate analysis		Multivariate analysis	
	HR (95% CI)	P value	HR (95% CI)	P value
Age (≥ 85/ < 85 years)	1.11 (0.22–5.63)	0.90		
Sex (male/female)	211421150 (N/A)	0.99		
Hernia localization (left/right)	1.78 (0.35–9.13)	0.49		
Manual reduction (failed/successful)	26.3 (3.04–227)	< 0.003**	23.9 (2.46–586)	0.005**
Time to symptom onset (≥ 72/ < 72 h)	15.0 (1.98–114)	< 0.009**	13.3 (1.20–345)	0.03*
ASA-PS score (2/3)	1.2 (0.21–6.80)	0.84		

The multivariate analysis included clinicopathological features with a P value of < 0.01 in the antecedent univariate analysis. *P < 0.05, **P < 0.01.

*N/A* not available, *ASA-PS* American society of anesthesiologists physical status, *CI* confidence interval, HR hazard ratio.

## Discussion

OH, which are rare, pose clinical challenges due to their anatomical location and presentation. Our study aimed to optimize the clinical approach for incarcerated OH by shedding light on the feasibility and efficacy of manual reduction prior to surgery, a topic that has been a matter of debate and has various applications across institutions. Results showed that the time to symptom onset is a significant predictor of bowel viability. This finding is in accordance with the guidelines on inguinal and femoral hernias^7^. Notably, patients with a time to symptom onset of ≥ 72 h were more likely to require bowel resection, possibly due to impaired circulation and consequent bowel necrosis. This result is similar that of previous studies showing that prolonged incarceration is a major risk factor of bowel nonviability^5,8^. Manual reduction was attempted in all cases in the current study. Despite these attempts, nine cases required bowel resection. This indicates that manual reduction might not be suitable for all patients, particularly those with the symptom duration of ≥ 72 h. These findings suggest the need for a more cautious approach toward patient selection for manual reduction; further revision of this protocol will be considered to reflect this insight.

Our previous study examined reduction techniques for OH^6^. However, the current study investigated the bowel viability predictors, which focused on factors such as time to symptom onset and outcomes of manual reduction. By including cases managed after 2017, the current study performed a more standardized assessment, thereby ensuring that all patients underwent manual reduction. Thus, our findings differed from the historical data used in the previous study.

Manual reduction, which is practiced at our institution, has a controversial association with bowel resection. Notably, failed manual reduction has emerged as a robust predictor of bowel nonviability, which requires resection. This result underscores the potential of manual reduction as not just a therapeutic procedure but a predictive maneuver, which indicates the severity of hernia incarceration and bowel compromise.

Further, the specificity of time to symptom onset was relatively low at 12%, which traditionally indicates limited ability to correctly identify patients without bowel nonviability. However, the high sensitivity at 67% and an AUC of 0.82 suggest that time to symptom onset is a robust predictor to identify patients at risk of bowel nonviability. These metrics emphasize that while time to symptom onset might not be as effective for excluding bowel viability cases (specificity), it is highly effective in identifying those at risk (sensitivity), making it a potential predictor in clinical decision-making.

Results showed that age, sex, hernia localization, ASA-PS score, and laboratory findings did not significantly differ between the bowel and nonbowel resection groups. This finding poses challenges in some traditionally held beliefs. It emphasizes the importance of individualized patient care where reliance on classical predictors may not always yield accurate prognostication.

The current study had several strengths. For example, it has a rigorous methodology, and it clearly differentiated patients based on their clinical outcomes. Further, it focused on an underrepresented clinical entity. However, it also had some limitations. That is, the sample size was modest; hence, the results should be validated in larger cohorts. Moreover, this was a single-center study. Thus, the generalizability of our findings might be limited, and multi-center studies should be performed in the future. Variation in the waiting period for elective surgery was another limitation of our study, which was influenced by external factors, particularly the COVID-19 pandemic. During the pandemic, hospital resources and scheduling were significantly affected, which led to inconsistencies in the timing of elective procedures. This variability might have affected the outcomes and should be considered while interpreting the results.

## Conclusion

Time to symptom onset and manual reduction outcomes play important roles in providing guidance regarding clinical decision-making for incarcerated OH. Manual reduction remains a viable initial approach, and patients with longer durations of incarceration and failed manual reductions should be evaluated promptly for surgical interventions due to a high risk of bowel nonviability. Nevertheless, future research should be performed to elucidate other clinical predictors, optimize management strategies, and reduce associated morbidities.

## Data Availability

The datasets analyzed during the current study are available from the corresponding author.
